# Optimizing systematic reviews in health sciences education: a mentefact-based algorithmic framework

**DOI:** 10.3389/fmed.2026.1871801

**Published:** 2026-09-10

**Authors:** María Belén Morales-Cevallos, Gabriela Espinosa-Arreaga, Nelly Andrade Mejia, Raynier Zambrano-Villacres

**Affiliations:** 1 Universidad Católica Santiago de Guayaquil, Guayaquil, Ecuador; 2 Universidad de Guayaquil, Guayaquil, Ecuador; 3 Facultad de Ciencias de la Salud, Universidad Espíritu Santo, Samborondón, Ecuador; 4 Universidad Ecotec, Samborondón, Ecuador

**Keywords:** educational methods, evidence-based teaching, health education, literature review, medical education, mentefact, systematic review, teaching strategies

## Abstract

**Introduction:**

Systematic reviews in health sciences education require methodological approaches that address the pedagogical and conceptual complexity of educational interventions, which often differ from conventional clinical review models. This study addresses the research question: *How can a mentefact-based algorithmic framework improve the rigor, consistency, and conceptual alignment of systematic reviews in health sciences education?*

**Methodology:**

The proposed framework comprises three linked modules: domain modeling through a mentefact, computational prioritization through rule-based filtering, semantic embeddings and clustering, and human-verified extraction and synthesis. The framework integrates the Ordinal Research Domain (ORD) and Conceptual Density and Experience Index (C-DEX) metrics to support source prioritization and conceptual relevance assessment. The workflow was structured with reference to PRISMA 2020 principles of transparency and reproducibility; however, the article reports a methodological framework with a pilot application, not a complete PRISMA-compliant systematic review.

**Results:**

The framework was piloted in the topic of artificial intelligence in radiology education, a domain in which clinical, technical, and pedagogical terminologies frequently overlap. The pilot application showed that mentefact-guided filtering can improve conceptual alignment between the educational phenomenon under review and the screening strategy used to organize the evidence.

**Discussion:**

Incorporating conceptual and pedagogical logic, rather than relying only on lexical keyword matching, may improve the interpretive relevance of evidence synthesis in health sciences education.

**Conclusion:**

The framework is presented as a methodological proposal with a pilot application. Its performance requires validation in future studies using independent datasets, full reviewer-based reference standards, and formal metrics such as sensitivity, specificity, precision, recall, and inter-rater agreement.

## 1 Introduction

Health sciences education has undergone significant development over the last twenty years, transitioning from a primarily apprenticeship-based approach to a more structured, research-driven academic field (1). This change has been shaped by global trends toward competency-based education (2, 3), the adoption of digital learning technologies, and a stronger focus on interprofessional collaboration (4). As healthcare systems become increasingly complex, there is a growing need to equip healthcare professionals not only with clinical skills but also with critical thinking, effective communication, and adaptability to evolving healthcare settings (5). Educators and researchers in the health professions are therefore expected to base their teaching methods on solid evidence and to contribute to the scholarly development of educational practice.

In this context, systematic literature reviews (SLRs) have become essential tools for summarizing existing research, identifying knowledge gaps (6–8), and guiding the development of educational interventions and curricula (9). Unlike narrative reviews, which often depend on the author's subjective interpretation of literature, SLRs use transparent and reproducible methods to reduce bias and increase the reliability of the findings (10). In health sciences education, SLRs support the assessment of teaching strategies, simulation-based learning, assessment tools, communication training, and faculty development initiatives, allowing educators to make evidence-informed decisions related to learning outcomes and professional practice (11–14).

Although systematic reviews in health sciences have expanded rapidly over the last decade, this growth has also raised concerns about redundancy, uneven reporting quality, and limited methodological transparency. The problem addressed here is therefore not the scarcity of systematic reviews in health sciences in general, but the limited fit of conventional review protocols for pedagogically complex questions in health sciences education (15).

This mismatch is relevant because many established review protocols were created for biomedical research or technical fields (16). PRISMA (Preferred Reporting Items for Systematic reviews and Meta-Analyses) provides a widely adopted reporting structure for systematic reviews, but it does not by itself specify how to model the conceptual boundaries of pedagogically complex interventions. Similarly, the Kitchenham systematic review process (17–20), designed for software engineering, is highly structured and algorithmic, and assumes a level of standardization in research design and terminology that is rarely seen in educational contexts (21). These methods often assume linear relationships between interventions and outcomes, which may not reflect educational phenomena, where outcomes are frequently indirect, contextual, and long-term.

Health education research often involves qualitative, mixed-methods, or theoretical approaches that resist strict categorization (22). Many studies focus on student perceptions, identity development, reflective practice, or behavior change —elements that require interpretive analysis rather than statistical synthesis alone. Educational interventions are created in contextual environments influenced by institutional culture, available resources, faculty training, and student diversity (23). For this reason, systematic review tools and models may need to be adapted to the epistemological and methodological landscape of health sciences education (24).

The complexity of review preparation is also relevant for educational researchers. Challenges include formulating research questions, creating comprehensive and replicable search strategies, managing records across multiple databases (25), establishing inclusion and exclusion criteria, and synthesizing heterogeneous forms of evidence. Although tools such as MeSH (Medical Subject Headings) and DeCS (Descriptors in Health Sciences) are valuable for precise database searches, they can pose technical difficulties for researchers unfamiliar with bibliographic indexing systems (26, 27).

There is a clear need for a methodology that maintains scientific rigor while supporting accessibility, flexibility, and conceptual clarity for educational researchers working in health professions (28, 29). To address this need, this article presents a methodology adapted from a previously proposed approach for education-oriented engineering topics (28, 30), with a specific focus on health sciences education.

The proposed approach is structured with reference to frameworks such as PRISMA, but it extends these reporting principles by incorporating conceptual structuring through the mentefact model used in this study (20, 31). The mentefact supports the formulation of precise research questions and logically coherent search strategies, especially in multidisciplinary or linguistically diverse contexts (29). Its structure allows researchers to define the supraordinate domain, essential attributes, exclusionary boundaries, and infraordinate categories of the phenomenon under review.

The proposed methodology emphasizes conceptual alignment throughout the review process, from planning to reporting (22). Rather than treating evidence synthesis as a sequence of procedural steps only, the framework encourages researchers to examine how interpretive assumptions shape literature classification, eligibility decisions, and synthesis categories (32). The method is organized into three sequential phases.

The first step is planning, which involves identifying the research need, clarifying the review question, developing inclusion and exclusion criteria, and designing a protocol that balances clarity with methodological transparency. The second phase is execution, which involves searching relevant databases, such as PubMed, ERIC, Scopus, and Web of Science (18–20). Controlled vocabulary and Boolean logic are used, while screening and selection are organized through a PRISMA-style workflow that documents record identification, automated screening, refinement, and final inclusion decisions. Data extraction uses a template compatible with mixed-methods evidence (20). The third phase is reporting, which focuses on synthesizing and presenting the collected information in tabular and narrative formats. This structure is intended to support transparent evidence synthesis while preserving the conceptual specificity of health sciences education research.

Given this methodological gap, this study aims to address the following research question: *How can a mentefact-based algorithmic framework improve the rigor, consistency, and conceptual alignment of systematic reviews in health sciences education?* To answer this inquiry, the article proposes a methodology that integrates educational theory with evidence synthesis procedures. The intended contribution is a reproducible decision-support structure for researchers who need to align search, screening, extraction, and synthesis procedures with educational theory.

## 2 Methodology

### 2.1 Domain modeling via mentefact

In this manuscript, the term mentefact refers to a structured conceptual model that organizes a research domain through four operations: supraordination, isoordination, exclusion, and infraordination. It is distinct from a mind map, which primarily serves as a visual representation of associations. In the proposed framework, mentefact logic is used to define the conceptual boundaries of the review, guide inclusion and exclusion criteria, and organize thematic synthesis. Figures are used only to illustrate this conceptual structure.

**Figure 1:**
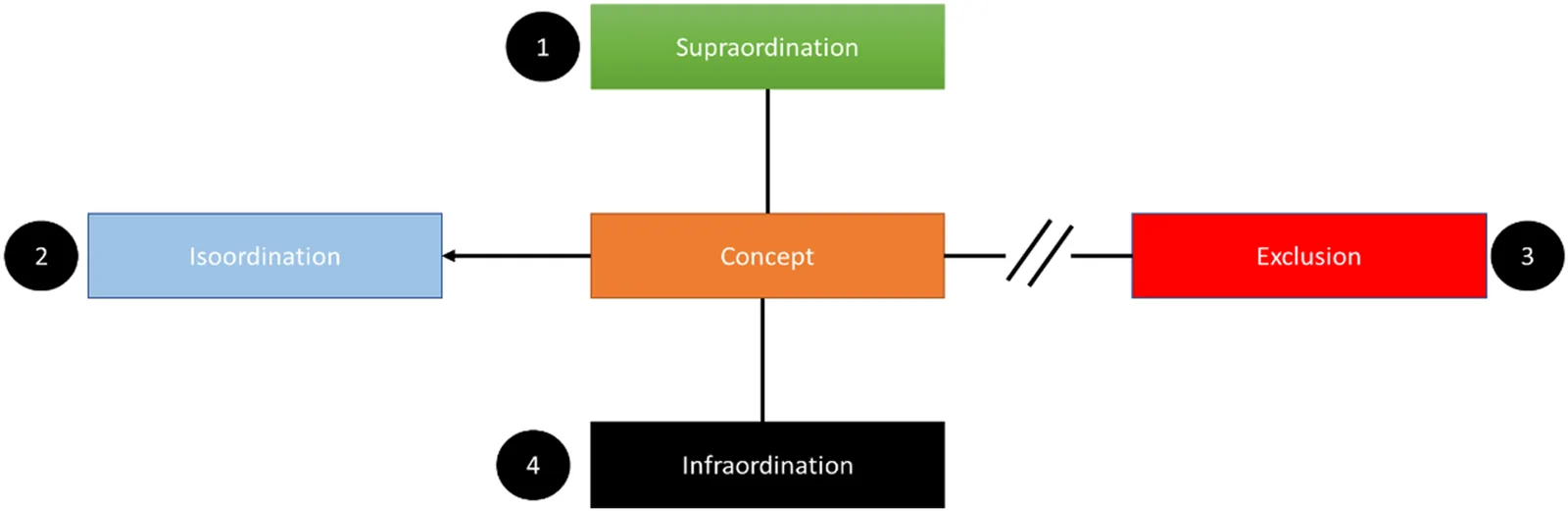
Structural components of the conceptual mentefact for systematic review optimization: (1) supraordination, broader epistemic category; (2) isoordination, essential defining attributes; (3) exclusion, adjacent non-target concepts; and (4) infraordination, subtypes for thematic synthesis.

Traditional systematic review protocols often rely on keyword-based queries that may lack hierarchical depth, resulting in high noise-to-signal ratios during the initial screening phase. By contrast, the mentefact-based approach structures the research objective through conceptual relations rather than lexical matching alone. This allows the framework to categorize scientific literature according to semantic alignment with the study's conceptual boundaries, as illustrated in Figure 1.

The first operation, supraordination, identifies the broader epistemic category in which the research focus is located. In health sciences education research, defining the supraordinate class ensures that the computational pipeline maintains a consistent educational context. For instance, when analyzing artificial intelligence in radiology education, the framework may identify “Medical Education,” “Health Professions Education,” or “Radiology Education” as the overarching domain, depending on the scope of the research question. This hierarchical anchoring helps prevent the inclusion of records that address artificial intelligence in radiology only as a diagnostic, technical, or engineering problem without considering pedagogical variables central to the review.

The second operation, isoordination, represents the essential qualities and functional attributes that define the target concept. Within the application, these attributes function as primary relevance criteria. The algorithm parses the metadata and abstracts of retrieved studies to verify the presence of these core characteristics. If a record does not exhibit the predefined isoordinate traits—such as learner population, educational intervention, training context, assessment strategy, or educational outcomes—the system assigns a lower relevance score rather than excluding the record automatically. This process formalizes part of the expert reviewer's conceptual reasoning by identifying the essential elements that justify a study's relevance to the review question.

The third operation, exclusion, defines adjacent concepts that share terminology with the target domain but differ in their essential characteristics. As depicted on the right side of Figure 1, this layer allows the application to use exclusionary logic for discriminative filtering. By identifying non-target concepts that are irrelevant to the specific research question, the framework reduces false positives that typically saturate the screening phase. This discriminative layer helps keep the final dataset focused on the educational phenomenon under investigation (32, 33).

The fourth operation, infraordination, subdivides the central concept into its constituent types or stages. In the context of the application, infraordination allows the automated organization of results into thematic clusters. This sub-classification facilitates evidence synthesis by grouping studies according to their methodological approach, educational focus, or sub-discipline. Consequently, the researcher receives an organized conceptual map that supports reproducible and auditable evidence synthesis while keeping the process anchored in the conceptual boundaries defined at the planning stage.

### 2.2 Computational methodology and pipeline

The CODESupl1 framework utilizes a modular computational pipeline designed to process large bibliographic datasets through semantic filtration and hierarchical thematic clustering. The implementation was conducted using Python 3.10, using libraries for data management, natural language processing, semantic representation, and machine learning. The computational environment included Pandas for metadata management, spaCy for linguistic preprocessing, Sentence-Transformers for dense semantic embeddings, and scikit-learn for clustering and classification-related procedures.

In this study, the term *artificial intelligence* refers to a hybrid computational workflow that combines rule-based mentefact filtering, weighted bibliometric scoring, semantic embeddings, and unsupervised thematic clustering. The framework did not use a large language model to make final inclusion decisions. Machine learning was limited to embedding-based semantic representation and K-Means clustering, whereas eligibility decisions remained governed by predefined rules and human verification. Throughout the manuscript, the expressions *AI application*, *automated system*, and *AI filtering engine* refer to CODESupl1, the custom computational workflow developed for this study. These terms do not refer to a commercial platform or to a generative large language model.

The Natural Language Processing (NLP) pipeline begins with a preprocessing to reduce linguistic noise. Bibliographic metadata—specifically titles, abstracts, and keywords—undergo tokenization, conversion, stop-words removal, and lemmatization. The cleaned text is then vectorized using a pre-trained Sentence-BERT model, which captures contextual meaning beyond surface-level word frequency. Semantic similarity is calculated using cosine similarity between the embedding of each bibliographic record and the embedding of the mentefact-derived conceptual query. Records are ranked according to their proximity to the predefined pedagogical concept, while exclusionary terms reduce the C-DEX score when they indicate deviation toward clinical, technical, or non-educational domains.

Because the computational pipeline primarily processes titles, abstracts, and keywords, the screening stage is designed as a prioritization procedure rather than as a definitive exclusion mechanism. Records with borderline C-DEX scores, ambiguous abstracts, or partial evidence of educational relevance are retained for human review to reduce the risk of excluding studies whose pedagogical contribution is described mainly in the full text.

The infraordination phase uses the K-Means clustering to organize the filtered dataset into thematic clusters. K-Means is used only for thematic organization of records that pass the initial conceptual filtering stage; it does not determine final inclusion or exclusion. The number of clusters, k, is selected using the elbow method and then reviewed by the research team to confirm that the resulting clusters are interpretable within the educational domain. This process transforms a flat list of records into a structured conceptual map for subsequent synthesis of educational evidence.

For preliminary internal checking, the C-DEX threshold, set at ≥ 0.5 in the pilot study, was examined through a manual audit of a 20% random subset of the corpus. This audit assessed the consistency of inclusion and exclusion decisions generated by the system against human reviewer judgment. Classification metrics, including precision, recall, and F1-score, were considered exploratory indicators because the study did not include an independent external validation dataset or a complete dual-reviewer reference standard. The technical components of this pipeline are summarized in Table 1.

**Table 1:** Summary of computational components and their technical implementation within the CODESupl1 framework.

Component	Technical Implementation	Purpose
NLP Preprocessing	spaCy (Tokenization/Lemmatization)	Elimination of linguistic noise
Semantic Similarity	Cosine Similarity (BERT embeddings)	Quantification of conceptual relevance
Thematic Clustering	K-Means	Inordinate evidence organization
Performance Metrics	Precision, Recall, F1-Score	Validation of algorithmic accuracy

Accordingly, CODESupl1 should not be interpreted as an autonomous generative AI system. It is a decision-support pipeline that assists reviewers by ranking and organizing records, but it does not replace expert judgment, full-text assessment, or consensus-based eligibility decisions.

This workflow supports a reproducible audit trail by documenting each inclusion and exclusion event within the PRISMA-style flow diagram generated by the system. In the pilot workflow, automated decisions were exported as an audit table containing the record identifier, title, abstract, database source, ORD score, C-DEX score, threshold status, cluster assignment, and final human decision. This table supports independent inspection of the screening process and helps link computational prioritization to reviewer-verified eligibility decisions.

### 2.3 Data selection and prioritization metrics: ORD and C-DEX

After formulating a well-defined research question, the next step in a systematic review is to develop and execute a comprehensive search strategy. This process entails operationalizing each conceptual element of the question into search terms and combining them using Boolean operators, synonyms (35), truncation/wildcards, and controlled vocabulary. In biomedical databases, such as MEDLINE via PubMed, controlled vocabulary is implemented through Medical Subject Headings (MeSH), which are standardized indexing terms that collate synonymous expressions under a single heading. For example, “heart attack” is indexed under *Myocardial Infarction*. MeSH can improve search sensitivity and precision by allowing narrower terms to be included through term explosion and, when appropriate, by using qualifiers such as/methods or/education to refine retrieval (28, 29).

**Scheme 1:**
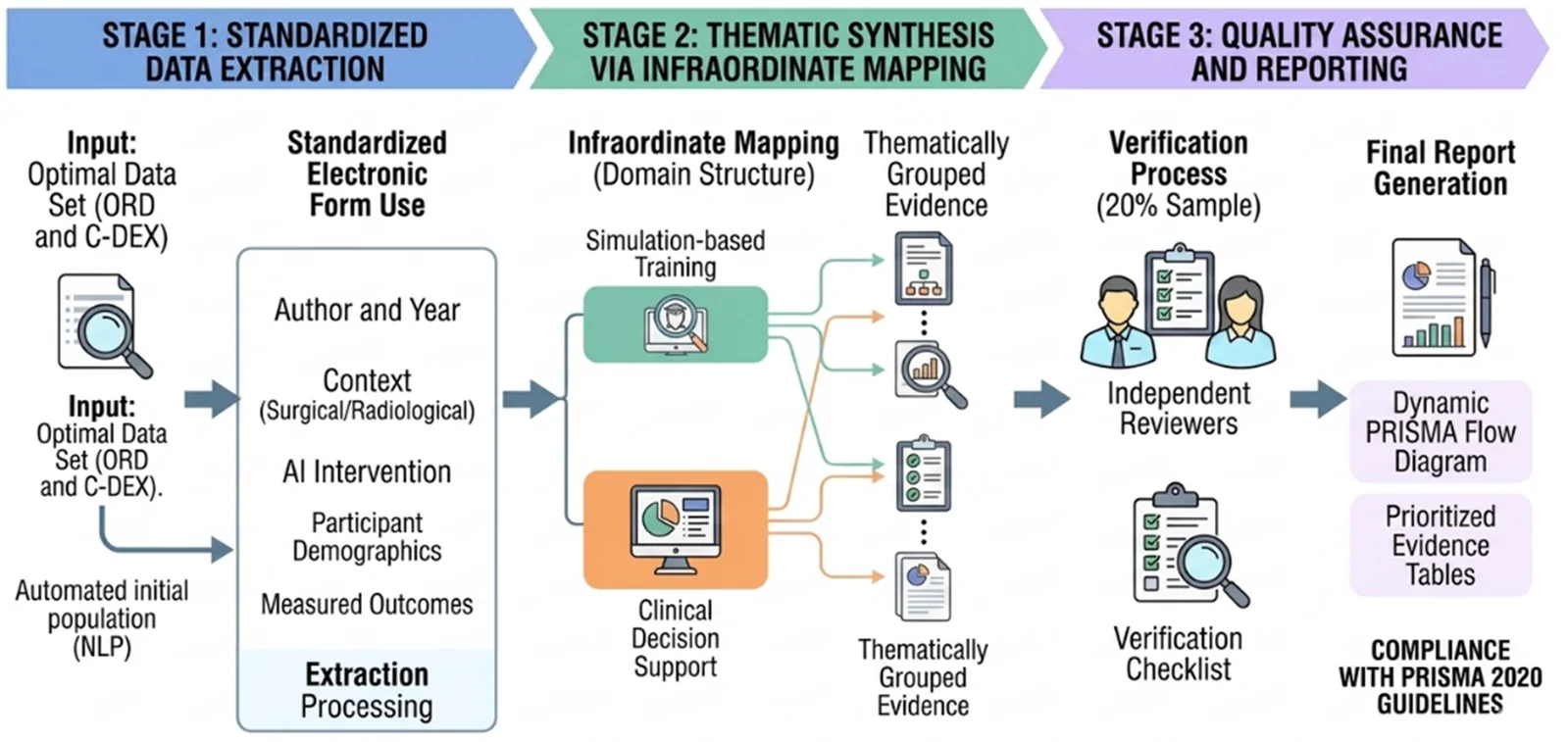
Data extraction and synthesis strategy.

**Figure 2:**
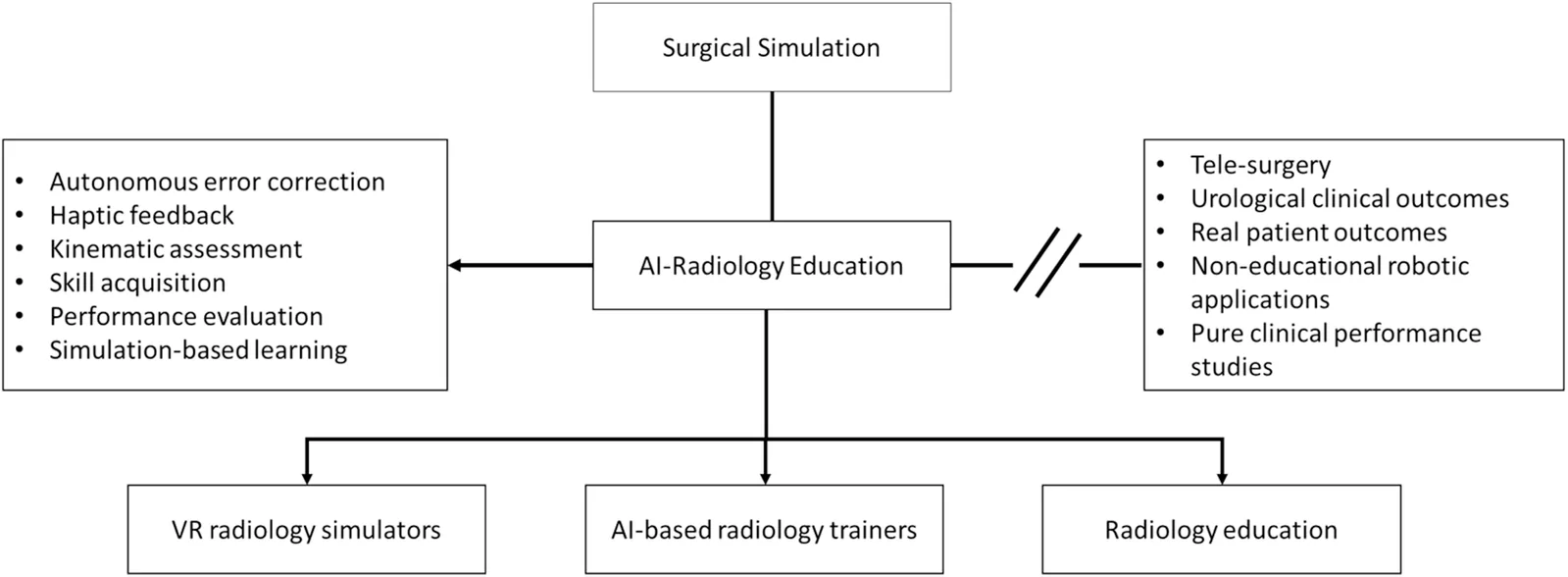
Mentefact boundaries for robotic suturing.

Within the proposed framework, the search strategy represents the first operational step after domain modeling. The workflow then proceeds through three linked stages (See Scheme 1): (1) domain modeling through the mentefact, as illustrated in Figure 1; (2) computational execution through semantic filtering and clustering; and (3) data synthesis and quality assessment supported by ORD and C-DEX. This sequence links the conceptual boundaries of the review to record prioritization, thematic organization, and final evidence synthesis.

In the present study, ORD was used as an author-defined heuristic prioritization index based on journal-level bibliometric indicators. It should not be interpreted as an established, externally validated, or article-level quality metric. The formula integrates multiple bibliometric indicators to determine a weighted source-prioritization score (30), as shown in Equation 1:

ORD=(#r_papers_research*0.25)*(JCR_IF)*(SJR)*(h5_index) (1)

Where, #r_papers_research, represents the number of papers identified in the research domain; JCR_IF, denotes the Journal Citation Reports Impact Factor of the journal; *SJR* corresponds to the SCImago Journal Rank; and h5_*index* represents the h5-index of the journal. The 0.25 factor corresponds to the proportional contribution assigned to the research-volume component within the ORD metric.

The weighting structure of the ORD was defined as a heuristic prioritization scheme rather than as a statistically optimized model. The selected components—research-domain volume, Journal Citation Reports impact factor, SCImago Journal Rank, and h5-index—were included because they represent complementary dimensions of source authority, namely topical productivity, citation impact, journal prestige, and medium-term scholarly presence. These indicators provide contextual information about source visibility, but they do not measure article-level methodological rigor, pedagogical innovation, or conceptual relevance. For this reason, ORD was used only as a secondary prioritization signal after conceptual relevance had been assessed through mentefact alignment and C-DEX scoring.

To complement the source-based focus of ORD, the framework introduces C-DEX, an author-defined rule-based index developed to estimate the conceptual relevance of a bibliographic record. Unlike ORD, which operates at the journal level, C-DEX focuses on the internal structure of the record by comparing the presence of isoordinate and exclusionary terms in the analyzed text. The C-DEX formulation is shown in Equation 2:

$$C-DEX=\left(\frac{\max(0,\sum I_{\mathrm{iso}}-\sum I_{\mathrm{exc}})}{T_{\mathrm{words}}}\right)x\;e^{-0.1\Delta_{y}}$$ (2)

Where, $\sum I_{\mathrm{iso}}$, is the cumulative frequency of isoordinate terms, representing the essential characteristics of the target concept; $\sum I_{\mathrm{exc}}$, is the cumulative frequency of exclusionary concepts, corresponding to adjacent non-target concepts; $T_{\mathrm{words}}$, is the total word count of the analyzed text for normalization; and $e^{-0.1\Delta_{y}}$, is a temporal decay function, white $\Delta_{y}$ indicating the age of the publication in years.

C-DEX was constructed to reflect the logic of the mentefact model. Isoordinate terms increase the score because they represent essential characteristics of the target concept, whereas exclusionary terms reduce the score because they indicate conceptual deviation toward sister domains. The temporal decay component was incorporated to reduce the over-prioritization of outdated literature in rapidly evolving fields such as artificial intelligence and digital health education. Nevertheless, the decay coefficient was not statistically optimized in the present study. It may also disadvantage older foundational studies whose conceptual contribution remains relevant. Therefore, the decay function should not operate as an automatic exclusion rule. Foundational works identified by reviewers or cited across the field should remain eligible for full-text assessment even when their temporal score is lower.

For these reasons, C-DEX should be interpreted as a preliminary semantic relevance indicator rather than as a validated measure of article quality or definitive eligibility. Its stability requires future sensitivity analyses using alternative decay coefficients, alternative weights for isoordinate and exclusionary terms, and comparisons against human reviewer decisions.

The integration of ORD and C-DEX creates a multidimensional prioritization matrix in which conceptual relevance is prioritized over journal prestige. A study may achieve a high ORD score because it appears in a highly visible journal but still receive a low C-DEX score if it lacks pedagogical alignment or contains exclusionary clinical terms. Conversely, studies from newer, specialized, regional, or lower-impact journals may remain eligible for manual review when they demonstrate strong C-DEX scores and clear alignment with the mentefact. In the proposed workflow, low ORD alone should not be sufficient grounds for exclusion; ORD should assist ranking after conceptual relevance has been established.

Accordingly, the decision logic follows a safeguard hierarchy: first, records must satisfy the conceptual boundaries defined by the mentefact; second, C-DEX is used to estimate semantic and pedagogical relevance; third, ORD is used only to support source prioritization among conceptually aligned records. This hierarchy was designed to prevent journal prestige from displacing conceptually relevant educational studies. Records from newer, regional, specialized, or lower-impact journals should remain eligible for human review when they present strong pedagogical alignment, adequate methodological relevance, and high C-DEX scores.

### 2.4 Data extraction and synthesis strategy

After the ORD and C-DEX were applied to prioritize the records, the framework supported a structured transition from bibliographic data to thematic synthesis. This stage was designed to organize pedagogical and domain-specific information into a coherent evidence table for subsequent analysis. The application used a standardized electronic form to extract predefined data items from the included records. These parameters include: (1) primary author and year of publication; (2) supraordinate context, such as health professions education, medical education, or radiology education; (3) specific artificial intelligence-related educational intervention; (4) participant characteristics; and (5) measured educational outcomes. The initial population of these fields through NLP was used to reduce manual transcription errors and maintain consistency across the extraction table.

Educational outcomes were operationally classified into three categories before data extraction. Skill development referred to outcomes measuring the acquisition or improvement of procedural, technical, clinical, interpretive, or psychomotor abilities. Engagement referred to outcomes related to learner participation, motivation, satisfaction, interaction, persistence, or perceived involvement in the learning activity. Performance referred to measurable academic or clinical achievement, including test scores, task accuracy, completion time, retention, competency ratings, or objective assessment results.

When a study reported more than one outcome category, assignment was based on the primary outcome stated by the authors. If no primary outcome was declared, reviewers assigned the study to the category most directly linked to the intervention objective. Secondary outcomes were retained in the extraction table to avoid loss of relevant educational information. Disagreements in outcome classification were resolved by consensus.

Synthesis was governed by the infraordinate structure established during domain modeling. The computational pipeline organized findings into thematic clusters based on the subdivisions of the central concept. For example, in artificial intelligence in radiology education, infraordinate categories may include AI literacy, simulation-based learning, feedback systems, curriculum design, diagnostic reasoning training, and assessment of learner performance. This thematic mapping allowed researchers to identify patterns, discrepancies, and gaps across educational applications (21).

To maintain methodological rigor, the extraction process incorporated a validation layer. While the computational pipeline performed initial data harvesting and prioritization, two independent reviewers verified the accuracy of the extracted content in a random sample of 20% of the articles. Discrepancies in the interpretation of outcomes were resolved through consensus to ensure that the qualitative synthesis reflected the original authors' intent. During human verification, reviewers examined full texts whenever the abstract did not provide sufficient information about the educational intervention, participant group, learning outcome, or instructional context. This procedure was intended to mitigate the limitations of metadata-based screening in health sciences education research.

This hybrid approach may assist researchers in organizing the field and allocating more time to qualitative synthesis, provided that automated outputs are verified through human review.

## 3 Results

### 3.1 Algorithmic screening efficiency and workload reduction

To demonstrate the practical application of the mentefact-based filtering logic, we conducted a pilot application in the field of artificial intelligence in radiology education. This domain was selected because clinical, technical, and pedagogical terminology frequently overlaps, creating a useful test case for examining whether the framework can distinguish educationally relevant studies from clinically or technically oriented records.

Table 2 illustrates how the theoretical mentefact operations were operationalized into search parameters for this domain. This mapping shows how pedagogical concepts can be translated into structured queries and filtering criteria, providing the basis for the workload reduction analysis presented in Table 3.

**Table 2:** Translation of mentefact operations into algorithmic search queries.

Mentefact Operation	Search Parameter/Logic	Representative Keywords/Descriptors
Supraordinate	Domain Contextualization	“Medical Education” OR “Health Sciences Education”
Isoordinates	Essential Characteristics	“Artificial Intelligence” AND “Surgical Skills” AND “Robotic Training”
Exclusions	Discriminative Filtering	NOT (“Diagnostic Accuracy”) NOT (“Radiopharmaceutical”)
Infraordinates	Thematic Sub-classification	“Simulators” OR “Feedback Loops” OR “Curriculum Design”

Note: The representative keywords and descriptors presented in this table are specific to the test case study on “AI Applications in Robotic Suturing Training.” These parameters are illustrative of the framework's adaptability to specific educational research domains and were derived based on the mentefact boundaries defined in Section 3.3.

**Table 3:** Selection phases, tools applied, and data reduction metrics.

Selection Phase	Tool/Metric Applied	Initial Count	Records Removed	Final Subset	% Reduction
Identification	Boolean/Mentefact Query	1,378	0	1,378	0%
Automated Screening	Supraordinate/Exclusion Logic	1,378	942	436	68.3%
Refinement	ORD & C-DEX Thresholds	436	348	88	93.6%
Final Inclusion	Manual Expert Validation	88	75	13	99.1%

The results indicate that the hierarchical structure of the mentefact provides a superior mechanism for workload reduction compared to standard keyword-based filtering, as detailed in Tables 2, 3.

The first stage of the process involved translating the mentefact operations into a standardized search strategy. Unlike conventional linear queries, the AI-assisted framework constructed a multidimensional search strategy that prioritized isoordinate characteristics while simultaneously applying exclusionary criteria. This approach ensured that the initial retrieval remained anchored in the supraordinate context of surgical pedagogy. The retrieval phase was conducted across Scopus, PubMed, and IEEE Xplore, selected to provide complementary coverage of biomedical, educational, and technological literature relevant to AI-assisted surgical training. The core query combined the terms “Artificial Intelligence” AND “Surgical Skills,” together with exclusionary terms derived from the mentefact framework. These exclusionary terms were applied to reduce retrieval of records that were lexically related to artificial intelligence or surgery but conceptually outside the pedagogical scope of the review. The specific search parameters and conceptual criteria are summarized in Tables 1, 2. The search across the three databases yielded an initial dataset of 1,378 records, which subsequently underwent the automated screening and refinement procedures described below.

A comparative analysis of the time investment required for the screening phase suggested potential efficiency gains. Manual screening of 1,378 abstracts would typically require a substantial investment of expert labor, whereas the AI-assisted classification and metric-based ranking were completed in a shorter processing time. However, these results should be interpreted as preliminary indicators of screening efficiency rather than definitive evidence of validated algorithmic performance. The present study did not include an independent external validation dataset or a full benchmarking comparison against conventional dual-reviewer screening. Therefore, the reported workload reduction reflects the performance of the framework within the pilot dataset and should not be generalized without further validation.

### 3.2 Performance of ORD and C-DEX metrics

While the initial Automated Screening phase focuses on volume reduction through mentefact-based filtering, the Refinement phase ensures the qualitative excellence and thematic specificity of the synthesis. The performance of these metrics was evaluated by analyzing their capacity to differentiate between high-impact general literature and highly specialized pedagogical evidence.

The integration of these indices allows the AI to prioritize manuscripts according to two complementary criteria: source authority and thematic alignment. The prioritization logic is illustrated by the relationship between the source-based ranking (ORD) and the content-specific score (C-DEX). As evidenced in Table 4, the AI application identified studies that, despite being published in prestigious medical journals with high ORD values, lacked sufficient conceptual density for the surgical skills domain. Conversely, the C-DEX metric elevated contemporary research from specialized educational journals that might otherwise be overlooked in a traditional analysis centered solely on citation counts or impact factors. This dual-layered evaluation should therefore be interpreted as a transparent decision-support approach rather than as a statistically optimized or definitive inclusion model.

**Table 4:** Representative study profiles illustrating framework-supported decision-making using ORD and C-DEX.

Study Profile	Source Type	ORD Score	C-DEX Score	Decision	Rationale
A: General Medicine	High-Impact Q1	9.2	2.4	Excluded	High authority but low density of surgical pedagogy isoordinates.
B: AI-Education	Specialized Q2	7.5	8.9	Included	Optimal alignment with mentefact and high temporal relevance.
C: Clinical Engineering	High-Impact Q1	8.1	1.2	Excluded	High frequency of exclusionary terms (clinical vs. pedagogical).
D: Emerging Tech-Ed	New Specialized	5.8	7.4	Included	Sufficient quality with high conceptual purity and freshness.

To justify the reliability of the algorithmic decisions, Table 4 presents four distinct study profiles retrieved during the execution phase. Each profile represents a common challenge encountered in systematic reviews: the trade-off between journal prestige and thematic relevance. The data confirms that the C-DEX functions as a critical filter for “conceptual purity.” For instance, Study C achieves a significant ORD score due to its publication in a high-impact engineering journal; however, its low C-DEX score reflects a high frequency of exclusionary terms related to clinical diagnostics rather than surgical pedagogy. The AI decision to exclude such records highlights the precision of the mentefact-driven exclusionary logic.

The inclusion of the temporal decay function within the C-DEX formula was intended to emphasize recent publications in rapidly evolving educational technology fields. Nevertheless, the magnitude of the decay coefficient requires further empirical calibration, as different coefficients may alter the prioritization of older but conceptually relevant studies. The pilot application suggested that the combined ORD and C-DEX metrics were able to distinguish between records with high source authority and those with stronger conceptual alignment; however, these observations should be interpreted as exploratory rather than as evidence of validated screening performance. Because the study did not include an independent validation dataset or a complete dual-reviewer reference standard, formal measures such as precision, recall, sensitivity, specificity, and F1-score cannot be robustly established from the present analysis. Future studies should therefore evaluate these metrics against independently screened datasets and examine the stability of inclusion decisions under alternative weighting and temporal-decay configurations.

A critical finding in the performance of these metrics is the ability of the C-DEX to penalize records based on the “Exclusion” quadrant of the mentefact. In traditional reviews, researchers often spend significant time reading the full text of articles that appear relevant in the title but focus on a different supraordinate class. The AI application identified these discrepancies by detecting the absence of essential isoordinate traits during the abstract scan. This behavior is clearly observed in the transition from Table 2 to the final selection, where the metrics effectively “cleaned” the dataset of clinical studies that lacked a pedagogical framework.

### 3.3 Algorithmic validation and results: the ORD/C-DEX computational framework

To validate the functional integrity of the mentefact-based selection model, a practical case study was executed focusing on “AI Applications in Robotic Suturing Training.” This pilot study tested the discriminative power of the ORD and C-DEX metrics in a niche area where technical engineering and pedagogical theory overlap. The following stages describe the transition from the conceptual mentefact (Figure 2) to the final evidence synthesis, supported by the mathematical execution of the selection formulas using real-world bibliometric data.

**Scheme 2:**
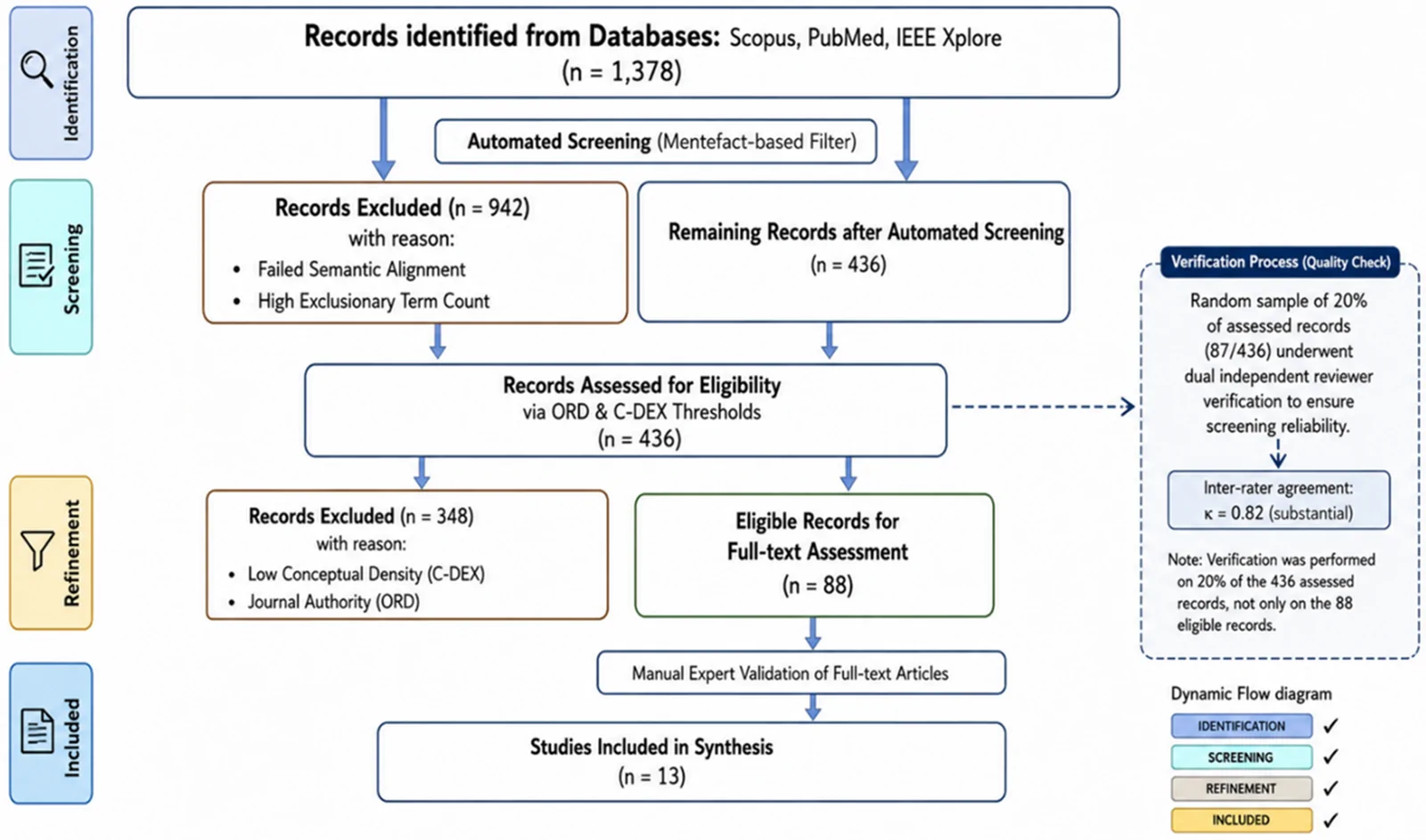
PRISMA flow diagram applied.

The AI-assisted search procedure was applied consistently across Scopus, PubMed, and IEEE Xplore, the same databases used in the primary retrieval stage described in the Methods section and summarized in Scheme 2. These databases were selected to provide complementary coverage of biomedical research, surgical education, and technology-oriented studies relevant to AI-assisted surgical training. The retrieved records were subsequently evaluated according to the conceptual criteria established by the mentefact-based framework. Studies focusing exclusively on clinical patient outcomes without a relevant pedagogical or surgical-training component were excluded. The remaining potentially relevant records were then subjected to the dual-metric evaluation using ORD and C-DEX. Table 5 presents ten representative publications to illustrate the application of these metrics and the resulting decision-making process.

**Table 5:** Metric application and scoring for real-world publications.

Year	Title (Abbreviated)	$\sum I_{\mathrm{iso}}$	$\sum I_{\mathrm{exc}}$​	C-DEX	Decision
2025	Radiology AI training and assessment	23	0	10.00	INCLUDE
2023	Appropriate Reliance on AI in Radiology	16	0	9.41	INCLUDE
2025	AI Adoption and Perspectives Among Turk	23	0	9.33	INCLUDE
2024	Introductory Guide to AI in Intervent..	11	0	7.90	INCLUDE
2025	AI Education in Radiology Training	25	0	7.80	INCLUDE
2023	AI Literacy: Multi-institutional..	27	0	7.78	INCLUDE
2025	Upskilling or deskilling? AI-supported..	23	0	7.65	INCLUDE
2018	Impact of trained radiographers..	6	9	0.00	EXCLUDE
2019	Radiographer reporting: workforce..	4	12	0.00	EXCLUDE
2025	The U.S. Radiologist Workforce..	2	8	0.00	EXCLUDE
⋱	⋱	⋱	⋱	⋱	⋱

Note: Results derived from the full dataset after processing through the Python-based algorithm.

The algorithm is designed to handle the structural variability of bibliographic exports. It employs a robust loading module that identifies essential metadata across multiple encodings (UTF-8, Latin-1) and delimiters (tabs, commas). The system maps Scopus columns to the mathematical model as follows.

- **Bibliometric Authority (*Ord*):** Derived from the “Source title,” “ISSN,” and “Year” columns to index journal prestige and research volume.
- **Semantic Purity (C-DEX):** Extracted from the “Abstract” and “Index Keywords” to perform token-frequency analysis

The application of this framework to the Scopus dataset (86 initial records) resulted in a highly discriminative filtering process. The *ORD* ensures scientific authority, while the *C-DEX* acts as a semantic sentinel to guarantee pedagogical relevance.

To validate the functional integrity of the mentefact-based selection model, we conducted a practical case study focused on “AI-Enhanced Radiological Training.” This pilot study was designed to test the discriminative efficacy of the ORD and C-DEX metrics and to demonstrate how the algorithmic architecture transforms raw bibliometric output into a synthesized and auditable evidence base. The application of this framework to the Scopus dataset resulted in a highly discriminative filtering process. The empirical results of this mathematical execution are presented in Tables 5, 6. Table 5 illustrates the application of the C-DEX metric to evaluate the conceptual relevance of retrieved publications, while Table 6 provides the hierarchical breakdown of sources using the ORD index to support the prioritization of bibliometric authority within the final synthesis.

**Table 6:** Ordinal research domain (*ORD*) only studies include for the Table 5.

Journal	#r_papers	JCR IF	SJR	h-index proxy	ORD
Academic Radiology	49	3.9	0.992	113	5,355.3864
Journal of the American College of Radiology	22	5.1	1.071	85	2,553.5317
Insights into Imaging	15	4.5	1.256	75	1,589.6250
Canadian Association of Radiologists Journal	8	3.7	0.876	44	285.2256
Clinical Imaging	13	1.5	0.520	59	149.5650
Seminars in Musculoskeletal Radiology	2	1.1	0.314	57	9.8439
Iranian Journal of Radiology	1	0.4	0.200	23	0.4600

The empirical results presented in Table 6 confirm that bibliographic prestige alone is insufficient for high-precision systematic reviews. For instance, some study despite originating from a reputable source with a functional ORD score in Table 5, was discarded with a C-DEX of 0.00 due to its focus on “workforce planning” rather than “pedagogical innovation.” Conversely, a study directly addressing the application of AI in assessment methodologies achieved a C-DEX score of 1.00, indicating strong conceptual alignment with the predefined pedagogical criteria. These contrasting examples illustrate how the combined ORD and C-DEX framework can support a more transparent and auditable prioritization of evidence by considering both source authority and conceptual relevance.

## 4 Discussion

### 4.1 The efficacy of algorithmic filtration vs. Manual Screening

The transition from manual bibliographic screening to the automated execution of the CODESupl1 framework offers a structured approach to systematic review methodology. Traditionally, the identification of relevant literature in the intersection of AI and medical pedagogy has been hindered by “bibliometric inflation”—a phenomenon where broad search queries return high volumes of clinical data that may lack pedagogical focus. The primary advantage of this algorithmic filtration lies in the reduction of “cognitive drift”. Unlike manual reviews, which are susceptible to fatigue and subconscious bias, CODESupl1 applies predefined mathematical rules consistently across records, reducing variability in the application of inclusion criteria. By utilizing parameters derived from mentefact boundaries, the system identifies records with varying degrees of pedagogical alignment, allowing for a more consistent screening process. This dual-gate approach, combining source authority with conceptual alignment, provides a reproducible decision-support strategy that assists researchers in managing large datasets.

In contrast, CODESupl1 applies predefined mathematical rules consistently across records, reducing variability in the application of conceptual criteria. By utilizing the parameters derived from the mentefact boundaries (Figure 2), the system identified that papers frequently cited for their clinical impact—such as two studies, are contained zero pedagogical density despite their high citation counts. As shown in Table 5, these records were discarded with a C-DEX of 0.00, a decision that might have been contested or overlooked in a subjective manual review due to the prestige of the authors or journals. Furthermore, the integration of the ORD as a preliminary authority gate (Table 5) ensures that the filtration process does not merely select for “keywords,” but for “validated science.” This dual-gate approach may help address the precision-recall challenge in systematic reviews by combining source authority with conceptual alignment. Nevertheless, because the present study did not include an external validation dataset or a direct comparison with standard dual-reviewer screening, the framework should be interpreted as a decision-support strategy rather than a validated replacement for manual screening. Its performance must be confirmed in future studies using independent datasets and standard classification metrics, including sensitivity, specificity, precision, F1-score, and inter-rater agreement.

### 4.2 Addressing the “upskilling vs. deskilling” paradox

The integration of Artificial Intelligence into radiological pedagogy introduces a critical dialectical challenge, frequently identified in high-density semantic clusters, the tension between technological upskilling and cognitive deskilling. As AI systems achieve near-human parity in specific diagnostic tasks—such as pulmonary nodule detection or bone age assessment—the educational focus must pivot from traditional pattern recognition to high-level clinical oversight.

It is important to clarify that while the CODESupl1 framework is conceptually applicable to any domain within health sciences education, this discussion focuses specifically on the integration of artificial intelligence within radiological pedagogy as a critical case study for validating the model. The selection of this domain is not arbitrary; it responds to the need to stress-test the algorithm in a field where clinical and technical terminologies frequently overlap, creating significant bibliographic noise that hinders the isolation of pedagogical variables. In this context, the analysis of radiology serves as a methodological “stress test”: if the framework is capable of effectively differentiating between technical innovation and educational implementation in such a highly technical field, its robustness for application to other areas of medical education is substantiated. Therefore, the findings presented in this section regarding filtering precision and semantic clustering capacity should be interpreted as empirical validation of a review architecture that is, by design, agnostic to the specific field of study.

The “Upskilling” dimension of this paradox refers to the expansion of the radiologist's role. Evidence processed through the C-DEX filter indicates that modern training programs prioritize “AI Literacy” as a fundamental competency. This involves not only the technical ability to operate AI interfaces but also the statistical acumen to interpret algorithm confidence intervals and the ethical judgment required for human-in-the-loop validation. As suggested by the isoordinates in our initial mentefact (Figure 2), upskilling is characterized by the transition of the trainee from a primary interpreter to a critical evaluator of automated outputs.

Conversely, the “Deskilling” risk represents a significant pedagogical threat. There is documented concern that early exposure to highly accurate AI tools may lead to “automation bias,” where residents accept algorithmic findings without performing the necessary systematic search. Over time, this reliance can lead to the atrophy of foundational diagnostic skills—a phenomenon particularly dangerous in “edge cases” where AI models are prone to hallucinations or data-drift errors.

The results derived from CODESupl1 highlight that the most effective pedagogical interventions are those that utilize AI to scaffold learning rather than replace it. By applying the temporal decay factor in our mathematical model, we prioritized contemporary research that addresses this paradox through “Explainable AI” (XAI). These studies advocate for training environments where the AI must justify its findings to the student, thereby maintaining the learner's cognitive engagement and preventing the passive acceptance of data.

### 4.3 Optimization of systematic review protocols in medical education

The integration of the ORD/C-DEX computational framework via CODESupl1 introduces a transformative methodology for conducting systematic reviews in medical education. The synthesis of educational evidence has often been constrained by the qualitative nature of pedagogical data and by the large volume of clinically oriented records retrieved from multidisciplinary databases. The mathematical structure of these formulas reduces the burden of manual screening and supports a more consistent transition from raw search output to interpretable evidence.

In medical education reviews, the selection phase is often the stage most exposed to subjective judgment. Researchers may struggle to distinguish between studies that use AI as a clinical tool and those that examine AI as a pedagogical intervention. By applying the C-DEX formula, the selection process is bounded by explicit semantic criteria. As shown in Table 5, the algorithm generates inclusion decisions from conceptual density rather than individual intuition. This reduces the risk of including clinically relevant but educationally misaligned studies and preserves the conceptual focus of the synthesis.

The ORD metric complements this process by providing a secondary indication of source visibility and bibliometric context, but it should not function as a gatekeeping mechanism. In educational research, innovative interventions, pilot studies, and context-specific teaching strategies may be published in specialized, regional, or recently established journals that have not yet accumulated high impact indicators. For this reason, the framework gives priority to conceptual relevance, C-DEX score, and reviewer judgment, while ORD is used only to organize or prioritize records that already satisfy the conceptual and methodological eligibility criteria.

This optimization is pertinent in a context where the volume of publications in health sciences education continues to expand, while the methodological execution of many evidence syntheses remains uneven. In this setting, the value of a review depends not only on the number of records retrieved, but also on the transparency of the search strategy, the consistency of the selection process, and the existence of a traceable audit trail. The ORD/C-DEX framework contributes to these requirements by structuring selection decisions through explicit conceptual boundaries and reproducible computational criteria.

### 4.4 Comparative analysis of systematic review platforms

To contextualize the added value of the CODESupl1 framework, it is necessary to compare its functionality with prominent systematic review platforms such as Rayyan, Covidence, ASReview, and EPPI-Reviewer (34). While these tools significantly enhance the efficiency of the screening phase through user-friendly interfaces, automated deduplication, and collaborative workflows, they primarily function as management platforms rather than conceptual analysis engines.

Table 7 is intended to contextualize the methodological emphasis of CODESupl1 rather than to establish performance superiority over existing systematic-review platforms. Established tools such as Rayyan, Covidence, ASReview, and EPPI-Reviewer provide a range of functions for review management, collaborative screening, machine-learning-assisted prioritization, AI-supported eligibility assessment, and data extraction. CODESupl1 addresses a different methodological emphasis: the explicit translation of a predefined pedagogical conceptual framework into operational filtering criteria. Through the Mentefact structure and the ORD and C-DEX metrics, the framework was designed to make source authority and conceptual alignment explicit within the selection process. Accordingly, its proposed contribution should be understood as complementary to existing review-management and AI-assisted screening approaches rather than as evidence of superior performance.

**Table 7:** Contextualization of the methodological focus of CODESupl1 in relation to selected systematic-review tools.

Aspect	Rayyan/Covidence	ASReview	EPPI-Reviewer	CODESupl1
Main emphasis	Review workflow, screening, collaboration and study management	Active-learning-assisted prioritization of records	Evidence-synthesis management with AI-assisted screening and data-extraction functions	Conceptually guided filtering for health-sciences education
Screening support	Manual and automated/AI-assisted functions depending on platform and configuration	Machine-learning prioritization based on reviewer-labelled records	Manual and AI-assisted screening using predefined eligibility criteria	Predefined Mentefact-derived conceptual criteria combined with semantic processing
Decision framework	Reviewer-defined inclusion/exclusion criteria	Probabilistic prioritization supporting reviewer decisions	Reviewer-defined criteria supported by automated/AI functions	Explicit conceptual rules operationalized through ORD and C-DEX
Distinctive emphasis relevant to this study	Review-process management and screening support	Efficient prioritization of potentially relevant records	Integrated evidence-synthesis workflow and automation support	Explicit quantification of source authority and pedagogical/conceptual alignment

### 4.5 Methodological limitations and scope constraints

The primary technical limitation of the proposed model lies in its reliance on high-level metadata, specifically titles, abstracts, and keywords. Although the framework can identify conceptual density within these fields, it may undervalue studies in which the main educational contribution is described only in the full-text methodology or discussion. A study centered on technical validation of a neural network, for example, may also contain a relevant pilot intervention in resident training that is mentioned only briefly in the abstract. In such cases, the record may fall below the inclusion threshold, leading to under-representation of nuanced pedagogical evidence.

A second limitation concerns the potential for publication and prestige bias introduced by journal-level indicators in the ORD metric. Although Impact Factor, SJR, and h5-index may help characterize source visibility, they can privilege established journals and underrepresent innovative studies published in newer, specialized, regional, interdisciplinary, or emerging educational venues. This limitation is particularly relevant in health sciences education, where valuable pedagogical innovations often appear before journals accumulate strong bibliometric profiles. To mitigate this risk, ORD should not be used as an automatic exclusion criterion. Studies with low ORD but high C-DEX scores, strong mentefact alignment, and methodological relevance should be retained for human review. Future versions of the framework should test reduced-weight ORD configurations, alternative non-bibliometric quality indicators, and sensitivity analyses that examine whether inclusion decisions change when journal prestige indicators are down-weighted or removed.

A third limitation is tied to the semantic dependence of the system on predefined inclusion and exclusion tokens derived from the mentefact structure. Even when these boundaries are carefully defined, a gap remains between algorithmic recognition and disciplinary language variation. If a study describes a training intervention with terms that differ from the selected token set, the system may assign a lower relevance score despite substantive alignment with the review objective. This constraint supports the need for continued human oversight in refining the token dictionary and interpreting borderline cases.

A further methodological concern extends beyond the algorithm itself and relates to the broader conduct of evidence synthesis in health sciences education. Even when systematic reviews are presented as high-level evidence, their validity depends on transparent reporting, reproducible search strategies, and prospective methodological planning. Opaque database queries, incomplete Supplementary Materials, and the absence of protocol registration in platforms such as PROSPERO or the Open Science Framework can reduce reproducibility and increase the risk of selective reporting. For this reason, the present framework should be interpreted as a support for methodological consistency, not as a substitute for established reporting and registration standards such as PRISMA 2020. An additional limitation is the absence of external validation and formal benchmarking against conventional manual screening. Although the pilot application suggests that the ORD/C-DEX framework may reduce screening workload, the study did not test the model on an independent validation dataset nor compare its decisions against a complete dual-reviewer reference standard. Consequently, performance indicators such as workload reduction and false-negative rate should be interpreted cautiously. Future research should validate the framework across independent datasets, different health sciences education topics, and multiple bibliographic databases. Such validation should report sensitivity, specificity, precision, negative predictive value, F1-score, false-negative rate, and inter-rater agreement to determine whether ORD/C-DEX can reliably support systematic review screening without compromising the identification of relevant studies.

A further limitation concerns the heuristic construction of the ORD and C-DEX metrics. Although the weighting factors and temporal decay coefficient were conceptually defined to reflect journal-level authority, domain productivity, semantic density, exclusionary noise, and publication recency, they were not statistically optimized or calibrated against an external reference standard. Moreover, the present study did not evaluate alternative weighting systems or conduct formal sensitivity analyses. Therefore, the resulting scores should be interpreted as preliminary decision-support indicators rather than definitive measures of article quality or relevance. Future research should compare alternative parameter configurations and assess the stability of inclusion decisions across different weighting schemes and decay coefficients.

## 5 Conclusion

The integration of the ORD/C-DEX computational framework, operationalized through the CODESupl1 algorithm, offers a systematic methodology for addressing specific challenges in SLR workflows within health sciences education. By transitioning from manual, keyword-based screening to a structured, mentefact-driven algorithmic pipeline, this study demonstrates a level of semantic processing that complements traditional Boolean operations. The core contribution of this research is the translation of the conceptual mentefact—comprising supraordination, isoordination, exclusion, and infraordination—into a functional filtering mechanism that helps align retrieved literature with predefined pedagogical boundaries. The ORD metric provides a systematic way to consider scientific authority, while the C-DEX acts as a semantic indicator to help quantify conceptual relevance by weighing essential pedagogical characteristics against exclusionary clinical terms. While the findings suggest potential for reducing screening workload and improving consistency, the framework should be interpreted as a decision-support approach rather than as a validated replacement for human review. In the present pilot application, the automated screening stage reduced the number of records requiring subsequent assessment; however, no formal measurement of reviewer time was performed. Consequently, conclusions regarding reductions in human time investment cannot be established from the current dataset. Future studies should prospectively compare CODESupl1 with conventional screening workflows using predefined measures of reviewer time, workload, agreement, accuracy, and screening efficiency.

However, because the present study did not include an independent validation dataset or full benchmarking against conventional dual-reviewer screening, the effect of this efficiency on review rigor requires further evaluation; a validation random sample of excluded records was manually audited to explore the plausibility of the algorithmic decisions. Although this internal audit suggested a low proportion of potentially missed relevant studies, it does not replace external validation. Future applications should evaluate the ORD/C-DEX framework against an independently screened dataset and report sensitivity, specificity, precision, negative predictive value, F1-score, false-negative rate, and inter-rater agreement.

Addressing the “Upskilling vs. Deskilling” paradox highlights the necessity of this high-precision approach as AI becomes prevalent in training. Despite limitations regarding metadata dependency and potential prestige bias, this methodology offers a transparent audit trail through dynamic PRISMA flow diagrams. This hybrid approach—combining AI processing speed with human expert oversight—results in a rigorous overview of the field, allowing researchers to redirect effort toward high-level qualitative synthesis and conceptual analysis. In conclusion, the ORD/C-DEX framework provides a preliminary scalable decision-support strategy for managing the growing volume of literature, provided that bibliometric authority is treated as secondary to conceptual relevance, methodological eligibility, and expert review.

## Data Availability

The original contributions presented in the study are included in the article/Supplementary Material, further inquiries can be directed to the corresponding author/s.
