# Supplementary material for: Optimizing systematic reviews in health sciences education: a mentefact-based algorithmic framework
Source: Front Med (Lausanne). 2026 Sep 10;13:1871801. doi: 10.3389/fmed.2026.1871801 (PMC13600972; doi:10.3389/fmed.2026.1871801)
Supplement: Supplementary file 1 [file Table1.docx]

**SUPPLEMENTARY INFORMATION: Optimizing Systematic Reviews in Health Sciences Education: A Mentefact-Based Algorithmic Framework**

**María Belén Morales-Cevallos^1,^*, Gabriela Espinosa Arreaga^2^, Nelly Mariel Andrade Mejia^3^, Raynier Zambrano-Villacres^4,^***

^1^Universidad Católica Santiago de Guayaquil, Ecuador

^2^Universidad de Guayaquil, Guayaquil, Ecuador

^3^Facultad de Ciencias de la Salud, Universidad Espíritu Santo, Samborondón 0901952, Ecuador

^4^Universidad Ecotec, Km. 13.5 Samborondón, Samborondón, EC092302, Ecuador

Corresponding authors: [maria.morales07@cu.ucsg.edu.ec](mailto:maria.morales07@cu.ucsg.edu.ec) (M.B.M-C) & [razambrano@ecotec.edu.ec](mailto:razambrano@ecotec.edu.ec) (R.Z-V)

1. **CodeSupl1.**

```
import pandas as pd
import re
import math
import os


def clean_text(text):
    if pd.isna(text):
        return ""
    text = str(text).lower().strip()
    text = re.sub(r"\s+", " ", text)
    return text


def count_tokens(text, token_list):
    total = 0
    for token in token_list:
        pattern = rf"\b{re.escape(token.lower())}\b"
        total += len(re.findall(pattern, text))
    return total


def read_scopus_file(full_path):
    encodings = ['utf-8', 'latin-1', 'cp1252']
    separators = ['\t', ',', ';']

    for enc in encodings:
        for sep in separators:
            try:
                df = pd.read_csv(full_path, sep=sep, encoding=enc)
                if len(df.columns) > 1:
                    print(f"[OK] File read using encoding={enc} and separator='{sep}'")
                    return df
            except:
                continue

    try:
        df = pd.read_csv(full_path, sep=None, engine='python', encoding='latin-1')
        print("[OK] Automatic reading applied")
        return df
    except Exception as e:
        raise ValueError(f"File could not be read properly. Detail: {e}")


def export_table_txt(df_res, output_path):
    with open(output_path, "w", encoding="utf-8") as f:

        f.write("SCOPUS VALIDATION RESULTS\n")
        f.write("=" * 100 + "\n\n")

        total = len(df_res)
        include = len(df_res[df_res["Decision"] == "INCLUDE"])
        exclude = len(df_res[df_res["Decision"] == "EXCLUDE"])

        f.write(f"Total studies: {total}\n")
        f.write(f"Included: {include}\n")
        f.write(f"Excluded: {exclude}\n\n")

        f.write("=" * 100 + "\n")
        f.write("FULL TABLE\n")
        f.write("=" * 100 + "\n\n")

        f.write(df_res.to_string(index=False))

        f.write("\n\n" + "=" * 100 + "\n")
        f.write("END OF REPORT\n")


def process_scopus_final():
    folder_path = r'Directory'
    file_name = 'File.csv'
    full_path = os.path.join(folder_path, file_name)

    print("\n--- Processing file ---")

    if not os.path.exists(full_path):
        print("[ERROR] File not found")
        print(os.listdir(folder_path))
        return

    try:
        df = read_scopus_file(full_path)
    except Exception as e:
        print(f"[ERROR] {e}")
        return

    print(f"\nTOTAL STUDIES LOADED: {len(df)}")

    # SCOPUS COLUMN MAPPING
    possible_columns = {
        "Abstract": ["Abstract", "Abstracts"],
        "Authors": ["Authors", "Author(s)"],
        "Year": ["Year", "Publication Year"],
        "Title": ["Title", "Document Title"]
    }

    columns = {}
    for key, options in possible_columns.items():
        for option in options:
            if option in df.columns:
                columns[key] = option
                break

    required_missing = [c for c in ["Abstract", "Authors", "Year"] if c not in columns]
    if required_missing:
        print(f"[ERROR] Missing required columns: {required_missing}")
        return

    col_abstract = columns["Abstract"]
    col_authors = columns["Authors"]
    col_year = columns["Year"]
    col_title = columns.get("Title", None)

    include_tokens = [
        'patient education',
        'ai',
        'chatbot',
        'training',
        'pedagogy',
        'radiology'
    ]

    exclude_tokens = [
        'workforce',
        'cancer strategy',
        'clinical trial',
        'policy'
    ]

    results = []

    for _, row in df.iterrows():
        abstract = clean_text(row.get(col_abstract, ""))
        authors = str(row.get(col_authors, "N/A"))
        title = str(row.get(col_title, "N/A")) if col_title else "N/A"

        year = pd.to_numeric(row.get(col_year, 2026), errors='coerce')
        if pd.isna(year):
            year = 2026
        year = int(year)

        i_include = count_tokens(abstract, include_tokens)
        i_exclude = count_tokens(abstract, exclude_tokens)

        total_words = len(abstract.split()) if len(abstract.split()) > 0 else 1
        delta_year = max(0, 2026 - year)

        c_dex_raw = (max(0, i_include - i_exclude) / total_words) * math.exp(-0.1 * delta_year)
        c_dex_norm = round(min(10, c_dex_raw * 100), 2)

        decision = "INCLUDE" if c_dex_norm >= 7 else "EXCLUDE"

        results.append({
            "Year": year,
            "Author": authors[:30],
            "Title": title[:60],
            "I_include": i_include,
            "I_exclude": i_exclude,
            "C-DEX": c_dex_norm,
            "Decision": decision
        })

    df_res = pd.DataFrame(results)

    print(f"TOTAL PROCESSED: {len(df_res)}")

    df_res = df_res.sort_values(by="C-DEX", ascending=False)

    print("\nRESULTS:\n")
    print(df_res.to_string(index=False))

    # EXPORT TXT ONLY
    output_txt = os.path.join(folder_path, "scopus_report.txt")
    export_table_txt(df_res, output_txt)

    print(f"\n[OK] TXT exported to: {output_txt}")


if __name__ == "__main__":
    process_scopus_final()
```

**Prompt Scheme 1**

Create a modern scientific workflow infographic with a clean white background and a professional academic design. The infographic should be organized horizontally into three sequential stages, connected by thick blue directional arrows, using a flat vector style with subtle gradients, rounded rectangles, and minimal shadows.

Stage 1 (Blue Header): STANDARDIZED DATA EXTRACTION

Large blue banner across the top labeled "STAGE 1: STANDARDIZED DATA EXTRACTION" in bold uppercase sans-serif font.

Left side contains two input blocks:

Magnifying glass icon labeled "Input: Optimal Data Set (ORD and C-DEX)"

Second identical input block beneath it.

Bottom text:

"Automated initial population (NLP)"

Center large rounded rectangle titled:

"Standardized Electronic Form Use"

Inside list:

Author and Year

Context (Surgical/Radiological)

AI Intervention

Participant Demographics

Measured Outcomes

Bottom label:

"Extraction Processing"

Stage 2 (Green Header): THEMATIC SYNTHESIS VIA INFRAORDINATE MAPPING

Green top banner labeled:

"STAGE 2: THEMATIC SYNTHESIS VIA INFRAORDINATE MAPPING"

Subtitle:

"Infraordinate Mapping (Domain Structure)"

Two stacked rounded boxes:

Top blue box with computer monitor icon:

Simulation-based Training

Bottom orange box with computer monitor and chart icon:

Clinical Decision Support

Multiple curved colored connectors flow toward the right.

Three clipboard/document icons on the right labeled:

Thematically Grouped Evidence

Include magnifying glass and analytics icons between connectors.

Stage 3 (Purple Header): QUALITY ASSURANCE AND REPORTING

Purple header labeled:

"STAGE 3: QUALITY ASSURANCE AND REPORTING"

Left section:

Verification Process (20% Sample)

Two reviewer avatars holding clipboards labeled:

Independent Reviewers

Additional clipboard with magnifying glass labeled:

Verification Checklist

Right section:

Document/report icon titled:

Final Report Generation

Three output boxes:

Dynamic PRISMA Flow Diagram

Prioritized Evidence Tables

Compliance with PRISMA 2020 Guidelines

Design Style

High-resolution vector infographic.

Medical research methodology aesthetic.

Flat illustrations with soft pastel blue, green, orange, and purple accents.

Rounded rectangles with light gradients.

Thin gray connector lines and thick blue process arrows.

Professional sans-serif typography (similar to Helvetica or Inter).

Consistent iconography with clipboard, magnifying glass, monitor, document, chart, reviewer, and analytics symbols.

Balanced spacing, clean alignment, minimalistic scientific journal figure style.

16:9 aspect ratio, ultra-high resolution (4K), crisp vector appearance, editable infographic aesthetic.

**Prompt Scheme 2**

Create a high-quality scientific PRISMA-inspired workflow infographic illustrating the study selection process for a systematic review. The design should be clean, publication-ready, and resemble figures published in high-impact journals (Nature, Elsevier, Springer, IEEE, MDPI). Use a white background, rounded rectangles with subtle gray borders and soft shadows, pastel-colored section labels, and thick blue downward arrows connecting every step.

The diagram should be organized vertically into four phases shown by colored vertical tabs on the left:

Identification (light blue)

Screening (light cyan)

Refinement (light orange)

Included (steel blue)

Top Section

Large centered rounded rectangle containing:

Records identified from Databases: Scopus, PubMed, IEEE Xplore (n=1,378)

Below it, a narrow horizontal box:

Automated Screening (Mentefact-based Filter)

Two large downward arrows branch into two boxes:

Left:

Records Excluded (n=942) with reason:

Failed Semantic Alignment

High Exclusionary Term Count

Right:

Remaining Records after Automated Screening (n=436)

Middle Section

Centered large rounded rectangle:

Records Assessed for Eligibility via ORD & C-DEX Thresholds (n=436)

Two downward arrows split into:

Left box:

Records Excluded (n=348) with reason:

Low Conceptual Density (C-DEX)

Journal Authority (ORD)

Right box:

Eligible Records for Full-text Assessment (n=88)

Inside the box include a highlighted pale-yellow rounded rectangle:

Verification Process: 20% Random Sample Audit

Bottom Section

A centered arrow beneath both branches with caption:

Manual Expert Validation of Full-text Articles

Leading to the final large centered rectangle:

Studies Included in Synthesis (n=13)

Bottom Right Corner

Small legend titled:

Dynamic Flow Diagram

Four colored rounded labels with checkmarks:

IDENTIFICATION (blue)

SCREENING (cyan)

REFINEMENT (orange)

INCLUDED (steel blue)

Design Style

Flat vector infographic

Professional academic layout

White background

Rounded rectangles

Thin gray outlines

Soft gray shadows

Thick blue process arrows with slight gradients

Consistent spacing and alignment

Bold sans-serif typography (Helvetica, Arial, or Inter)

Publication-quality graphical abstract

Modern PRISMA-inspired methodology figure

Clean minimalistic scientific visualization

Soft pastel blue, cyan, orange, and gray color palette

Ultra-high-resolution vector appearance (4K, 16:9 aspect ratio)
